# Real-Time Visual Feedback Based on MIMUs Technology Reduces Bowing Errors in Beginner Violin Students

**DOI:** 10.3390/s24123961

**Published:** 2024-06-19

**Authors:** Cecilia Provenzale, Francesco Di Tommaso, Nicola Di Stefano, Domenico Formica, Fabrizio Taffoni

**Affiliations:** 1 Advanced Robotics and Human-Centred Technologies–CREO Lab, Università Campus Bio-Medico di Roma, 00128 Rome, Italy; c.provenzale@unicampus.it (C.P.); f.ditommaso@unicampus.it (F.D.T.); f.taffoni@unicampus.it (F.T.); 2 Department of Electrical and Information Engineering, University of Cassino and Southern Lazio, 03043 Cassino, Italy; 3 Institute of Cognitive Sciences and Technologies (ISTC), National Research Council of Italy (CNR), 00196 Rome, Italy; nicola.distefano@istc.cnr.it; 4 Neurorobotics Lab, School of Engineering, Newcastle University, Newcastle Upon Tyne NE1 7RU, UK

**Keywords:** bowing angles, inertial measurement unit, real-time visual feedback, usability, violin beginners

## Abstract

Violin is one of the most complex musical instruments to learn. The learning process requires constant training and many hours of exercise and is primarily based on a student–teacher interaction where the latter guides the beginner through verbal instructions, visual demonstrations, and physical guidance. The teacher’s instruction and practice allow the student to learn gradually how to perform the correct gesture autonomously. Unfortunately, these traditional teaching methods require the constant supervision of a teacher and the interpretation of non-real-time feedback provided after the performance. To address these limitations, this work presents a novel interface (Visual Interface for Bowing Evaluation—VIBE) to facilitate student’s progression throughout the learning process, even in the absence of direct teacher intervention. The proposed interface allows two key parameters of bowing movements to be monitored, namely, the angle between the bow and the string (i.e., α angle) and the bow tilt (i.e., β angle), providing real-time visual feedback on how to correctly move the bow. Results collected on 24 beginners (12 exposed to visual feedback, 12 in a control group) showed a positive effect of the real-time visual feedback on the improvement of bow control. Moreover, the subjects exposed to visual feedback judged the latter as useful to correct their movement and clear in terms of the presentation of data. Although the task was rated as harder when performed with the additional feedback, the subjects did not perceive the presence of a violin teacher as essential to interpret the feedback.

## 1. Introduction

Learning how to play the violin is a long process that requires many hours of study and practice [1,2]. Indeed, it has been estimated that 700 h might be needed to learn the basics of bow control, i.e., to perform regular and fluid movements that allow for the emission of a sound with sufficient timbral quality [3]. 

The learning process of a complex motor task can be divided into four phases [4,5] depending on the level of awareness and proficiency of the student, in particular: (i) unconsciously unskilled; (ii) consciously unskilled; (iii) consciously skilled; and (iv) unconsciously skilled. In the first phase, the students are not conscious of their mistakes. The teacher’s feedback allows the students to achieve awareness of their errors, even if they are still not able to execute the task correctly (i.e., they move to the second step of the learning process). Continuous practice allows students to achieve the third phase, in which they can execute the exercise while correctly focusing on it. Finally, with consistent practice and teacher’s instructions, students acquire the ability to execute the task with automatized movements. Teachers play a crucial role in the student’s progression through the different phases of the learning process. The presented four-phase learning scheme has also been applied to the study of violin [6]. In the first two phases, teachers provide students with hands-on guidance by physically directing their gestures. As teaching progresses to more advanced stages, the students are exposed to visual demonstrations by the teachers and requested to replicate the observed movements. Teachers evaluate students’ performance and provide feedback, allowing them to gain awareness of their mistakes. When the students have learned how to master the violin (unconsciously skilled), they can concentrate their effort on more complex aspects related to interpretation, such as how to play music expressively. 

Unfortunately, the traditional teaching method currently employed is prone to some limitations. Being primarily based on physical student–teacher interaction, this training approach requires continuous teacher supervision. Moreover, the student may be unable to correctly interpret the teacher’s verbal instructions and physical guidance, which are often offered at the end of the performance (i.e., delayed feedback) [7]. For example, after playing an exercise, the teacher might point out incorrect movements during bow changes, suggesting that the student relax their shoulder or avoid exerting unnecessary resistance or effort when changing the direction of the bow. Such qualitative instructions can be unclear regarding how to implement them precisely in the exercise, and the trainee might struggle to translate them into practice.

To overcome these limitations, the use of new smart interfaces recording the bowing gestures and providing students with quantitative feedback could be explored. These new interfaces may enable an augmented information flow useful to monitor the bowing gesture and to provide real-time feedback to students. In the literature, several studies have investigated the use of different types of feedback to support violin learning. In [8], the authors designed a wearable system (i.e., MusicJacket) that works in combination with an optical motion capture system to provide vibrotactile feedback to students. This work highlighted the advantages of such feedback for improving some aspects of bow control, such as bow stroke length and bowing straightness, as well as the holding position of the instrument (i.e., the correct positioning of the violin near the neck). However, the results pointed out the influence of vibrotactile feedback on the students’ cognitive load, increasing the overall difficulty of the exercise. Moreover, the authors underlined the significance of the teacher’s presence for accurately interpreting the feedback, since the MusicJacket identifies deviations from the target movement but does not provide guidance on corrective measures. Furthermore, being a wearable system, the proposed solution has to be adapted to different anthropometries (see also [9]). Grosshauser tried to overcome this limitation by placing two vibrotactile motors on the bow frog [10]. However, no data on the students’ perception have been collected in support of the modality proposed to render the vibrotactile feedback. A different approach was proposed by Ng et al. [11], who developed a graphical interface (namely, i-Maestro) for a 3D representation of the student’s performance by using an optical motion capture system for data acquisition and gesture reconstruction. i-Maestro allows for the visualization of different bowing parameters (e.g., bow–bridge distance and bow angles) in the form of a graph. The authors also employed audio feedback to alert students of their mistakes, but it was perceived as interfering with the music performance. Moreover, this system only pointed out the occurrence of mistakes, without providing students with any suggestions on how to correct them. Blanco et al. proposed the use of visual feedback [12], a motion capture system (i.e., a Microsoft Kinect), and a microphone to record the sound. The integrated system, namely, SkyNote, provides visual feedback on the quality of the sound and on the bow movement reconstructed from the kinematic data. The quality of the sound is assessed in terms of pitch and timber, while the bowing movements are reported as 2D visual representations of quantitative parameters, such as bow inclination, tilt, and skewness, as well as the bow–bridge distance. No information on the correctness of the movement is provided. The results obtained from six training sessions showed improvement in the students exposed to visual feedback with respect to the control group. Nevertheless, the authors emphasized that the additional feedback adversely affected the cognitive load. Indeed, the improvement of the bowing movements paralleled the loss of quality sound.

In line with the reviewed literature, in this work, we aim to investigate the effectiveness and usability of a smart feedback-based system in promoting error awareness in violin beginners, also without the physical presence of a teacher. The following criteria have been considered to assess each type of feedback and to guide its selection: (i) the necessity to apply external devices on the beginner’s body (referred to as obtrusiveness); (ii) the necessity of trained personnel to interpret the feedback; (iii) the effectiveness of the feedback; and (iv) the impact of feedback on the cognitive load (i.e., the amount of information involved in the task execution that an individual can process and manage at any given time [13]). The criteria are derived from literature works in which different types of feedback have been tested. Table 1 briefly summarizes the assessment described in detail below.

The results reported in the literature on vibrotactile feedback showed an overall improvement in the bow performance, but at the expense of the cognitive load [8]. Moreover, this approach showed limits in its usability, requiring the adaptation of the system to the user’ body and continuous teacher supervision for a correct interpretation of the feedback. The audio feedback proposed by [11] did not show these limitations, but was not effective in guiding the students [8]. On the other hand, the visual feedback proposed by [12] showed positive outcomes on the improvement of bowing gestures at the cost of additional cognitive load. Nevertheless, this latter approach does not require the application of external devices on beginners’ body nor the presence of a teacher, which makes it the most promising. Considering the usability, all reviewed works [8,11,12] proposed the use of an optical motion capture system for the acquisition of the bowing gesture. This requires a non-trivial preparation of the environment to mitigate reflection issues and, in several cases [8,11], the use of optical technology, which may prove costly for large-scale implementation.

This work presents the design and development of an interface that supports violin training, providing students with real-time feedback that attempts to trade off between efficacy and the usability of the technology exploited for gesture monitoring. The interface leverages on the use of visual feedback in combination with magnetic inertial measurement units (MIMUs) for bow-angle monitoring [14], developing a system for the real-time assessment of the task. Given that, in the first year, of practice students typically learn how to properly control the bow through open-string exercises (i.e., without using the fingerboard), this work focuses on providing real-time measures of the bow–violin orientation and feedback to correct possible mistakes during open-string exercises.

## 2. Materials and Methods

**Figure 1:**
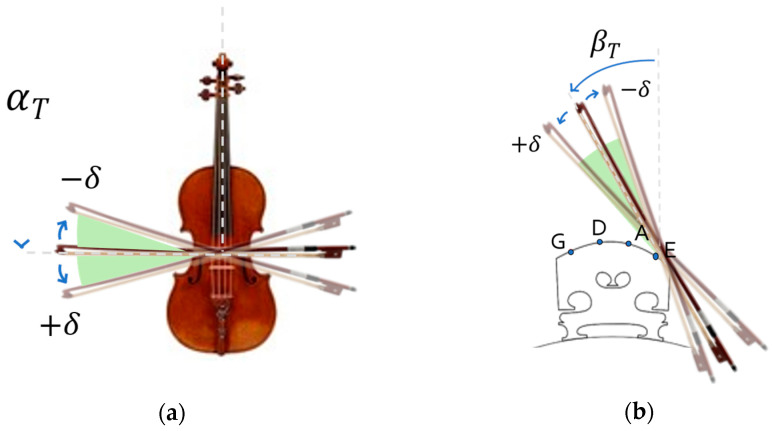
Bowing angles: (**a**) α angle, the angle between the bow hair and the violin string; (**b**) β angle, tilt of the bow with respect to the axis normal to the sound board (focus on the violin bridge, sketch not in scale).

The assessment of the bow–violin orientation was performed by monitoring two angles: (i) the angle between the bow hair and the violin string, measured in the plane parallel to the violin sound board (i.e., α angle), and (ii) the bow tilt (i.e., β angle) (Figure 1).

The correct execution of the exercise requires keeping the bow parallel to the bridge and playing one string at a time. For this reason, the α angle should be kept approximately at 90° with respect to the strings, i.e., $\alpha_{T}\pm \delta$ (Figure 1a). The target value of the β angle ($\beta_{T}$, Figure 1b) depends on the string played and may vary within a range. A feasibility study with an expert musician [14] allowed these angles to be derived, and a correlation analysis was performed between the string played and the bow tilt. For the α angle, the range was set to ±10° according to the opinion provided by the expert musician (i.e., third author) involved in the study. For the β angle, the angular range around the target orientation was set as the bisector of the difference between the target angles of two adjacent strings. When a string had more than one neighbor (as for the central strings D and A), the minimum bisector value was considered. For example, for the A string, the range interval was estimated as:

(1) $$\delta_{A}=min\left(\frac{\left|\beta_{T,A}-\beta_{T,D}\right|}{2},\;\frac{\left|\beta_{T,A}-\beta_{T,E}\;\right|}{2}\;\right)$$

Table 2 reports the $\beta_{T}$ values measured for each string and the corresponding angular ranges (±δ).

**Figure 2:**
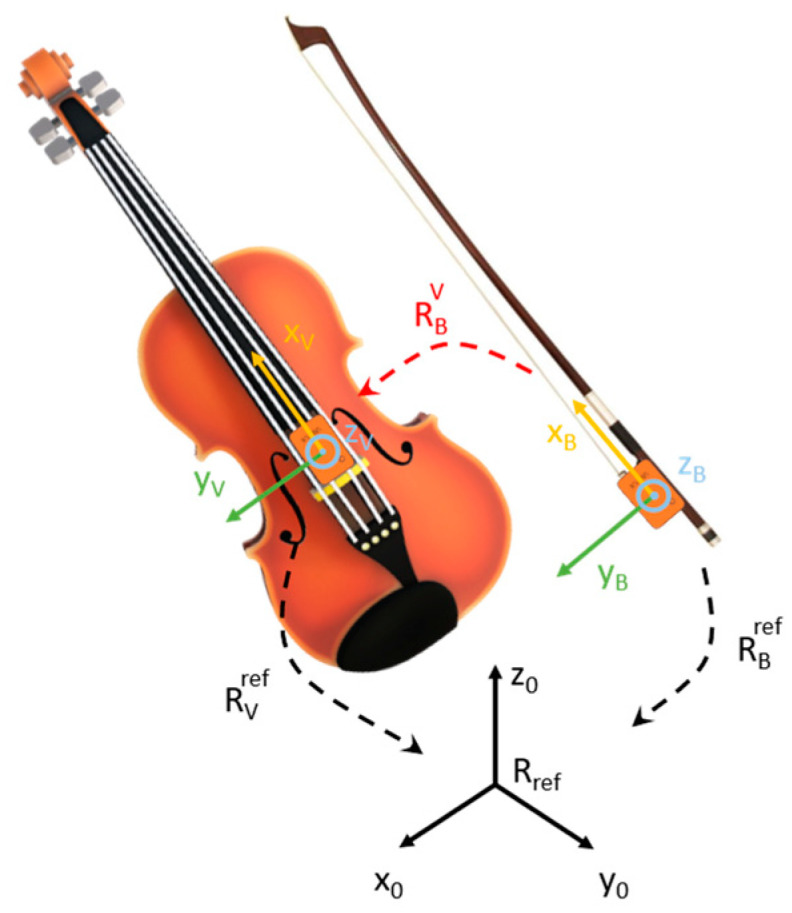
Placing of MIMU sensors on bow and violin. $R_{V}^{ref}$ and $R_{B}^{ref}$ express violin and bow orientation with respect to the reference triad ($R_{ref}$), represented in black, respectively. $R_{B}^{V}$ expresses the orientation of the bow with respect to the violin triad.

Based on the results described in [14], two Xsens MTw Awinda (Movella Inc., Henderson, NV, USA) magneto-inertial measurement units were used. These sensors estimate orientation by exploiting a proprietary sensor fusion algorithm (i.e., the Xsens Kalman Filter) for human motion [15]. The sensors were aligned with a custom-made support, placed on a table, and kept still for about 30 s to ensure settling time [15]. A heading reset operation was performed to guarantee the alignment of the local earth reference frame of each sensor and compensate for possible noise sources, as in [14]. An additional source of error may be orientation drift [16]. This problem is particularly evident in the case of rotation along the gravity axis: in this case, the sensor fusion algorithm heavily relied on magnetic field measurements, and in the presence of a perturbation, no reference measure at all was available to stabilize drifts on the heading component of the orientation [17]. For this reason, tests were performed in a quiet room away from ferromagnetic objects and known sources of magnetic fields (excluding the laptop used for the acquisition). After the warmup time [16], one sensor was placed on the frog of the bow with the *x*-axis directed toward the bow tip, while the second one was applied on the violin between the fingerboard and the bridge, with the *x*-axis oriented toward the pegbox and with the *z*-axis perpendicular to the soundboard (Figure 2). Knowing the relative orientation of the bow with respect to the violin body, it was possible to estimate the bowing angles (i.e., *α* and *β* angles) (see [14] for more details on the method applied). The Xsens MTw Awinda software 4.6 development kit (SDK) was used for: (i) managing the wireless (i.e., Bluetooth) communication with MIMUs; (ii) initializing the sensors; (iii) managing the synchronization between the two MIMUs; and (iv) performing a heading reset operation to provide the sensors’ orientation with respect to a common reference frame.

### 2.1. Visual Interface for Bowing Evaluation

The sensor orientations expressed in quaternions were collected and streamed in real time through a UDP connection from the Xsens MTw Awinda SDK to a specifically developed graphical user interface, namely, the Visual Interface for Bowing Evaluation (VIBE). VIBE is designed to allow (i) the real-time estimation of bowing angles (i.e., α and β angle); (ii) the generation of visual feedback; and (iii) data storage of quaternions (i.e., raw data) and bowing angle data. VIBE was designed through a combination of Python language and the QtDesigner 4 tool. The latter was used to build the graphical widgets of VIBE.

**Figure 3:**
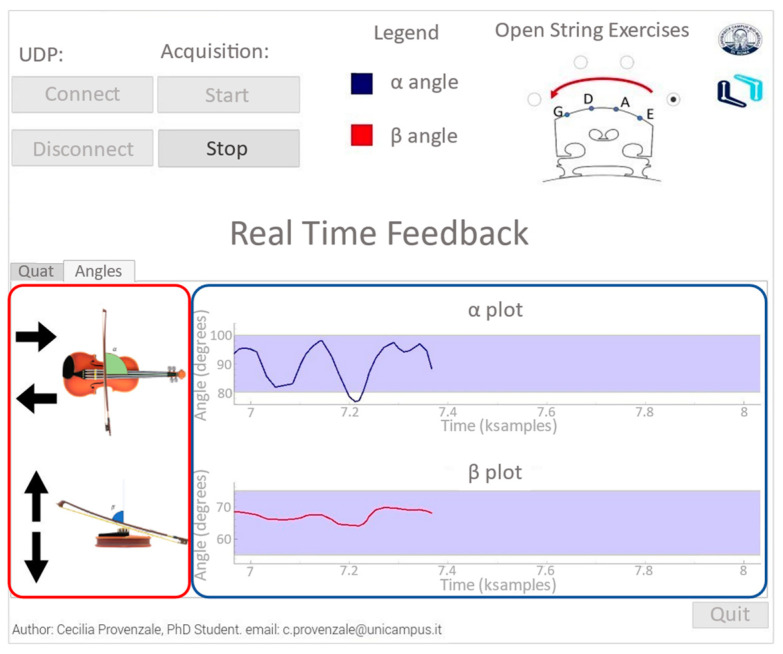
VIBE dashboard: the upper section allows the recoding and set configuration parameters, such as the string played, to be managed. In the bottom part, real-time feedback is provided. Plots in the blue box show the bowing angles and the violet rectangles shows the target intervals in which the movement can be considered as correct. Icons in the red box provide user with suggestions on how to correct their movement. The Tab Quat provides the raw orientations of the sensors in quaternions, and it is used only for debug purposes.

Figure 3 shows the main VIBE dashboard. The upper part is devoted to configuration purposes: users can enable the UDP communication, select the desired open-string exercise, and start/stop the training session. In the bottom part of the window, users can visualize raw data or the bowing angles in real time and concurrently receive visual feedback on the target angles and the optimal range of movement for a correct execution of the exercise. Two violin icons with two black arrows each are shown on the left side of the real-time feedback tab (one icon for each bowing angle). If the user exceeds the target interval, the interface suggests how to adjust the bow orientation by highlighting the arrow pointing in the direction necessary to correct the movement. For example, when α>90°+δ (i.e., 100°), the upper arrow pointing on the right will be highlighted. For the β angle, the target value and its range depend on the string played, which can be selected via the open-string exercise section. This function changes a flag on the exported file that could help students with a successive offline additional analysis of their performance. Indeed, VIBE generates as an outcome a file for each MIMU containing the quaternions and a file with the processed data. Additionally, each file contains information on the string played.

### 2.2. Participants and Procedure

**Figure 4:**
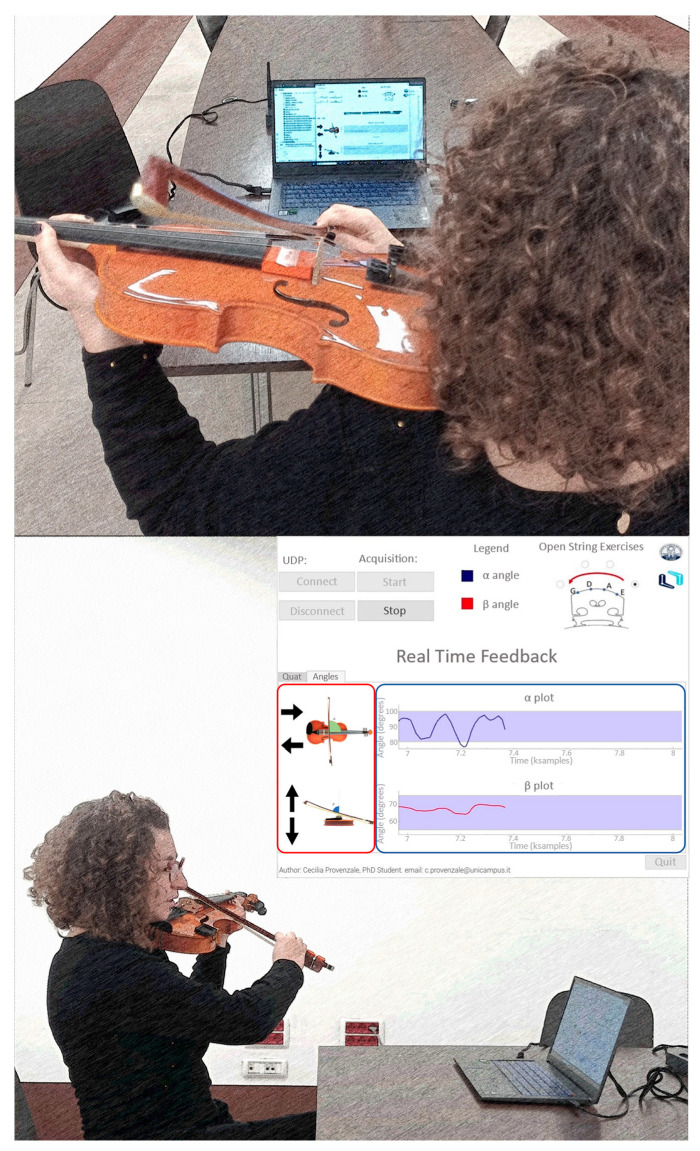
Experimental setup. Participants in the F group performed the exercise with real-time visual feedback.

Twenty-four beginners (10 females) aged 27.96 ± 3.90 years (mean ± SD) were enrolled in the study. They had normal or corrected-to-normal vision (11 participants wore glasses), and they were all right-handed. This study was preliminarily approved by the ethics committee of the Università Campus Bio-Medico di Roma (protocol code PAR 25.21, Rome 31 March 2021), and informed consent was obtained from all subjects involved in the study. Participants were requested to comfortably sit on a chair. They were provided with a violin and a bow instrumented with the MIMU sensors and asked to perform simple open-string exercises in a two-phase protocol. During the first phase (i.e., the baseline phase), participants were requested to repeat 10 strokes for each string at their best without any constraint on the tempo. In the second phase (i.e., the experimental phase), 12 subjects were provided with visual feedback (F group) through a laptop running VIBE which was placed in front of the participants (Figure 4). The remaining 12 subjects were registered without exposure to the feedback (No Feedback (NF) group). Both groups were asked to perform 10 strokes on each string, repeating the exercise at three different tempi using a metronome: 60 bpm (slow tempo), 80 bpm (medium tempo), and 100 bpm (fast tempo). In both phases, the bowing angles and the angular error (difference between the measured and the target angle) were estimated. The absolute values of these errors were subsequently averaged along each string to obtain a mean absolute error (MAE). The MAEs were compared between groups to test the initial group skill (MAE measured in the baseline phase) and the efficacy of the correction allowed by the real-time visual feedback provided to the F group in the experimental phase.

Additionally, to assess the subjects’ experience of the system as well as their perception of the real-time visual feedback, the participants in the F group were requested to complete the System Usability Scale (SUS) [18] and an online questionnaire on the visual feedback. The questionnaire (see Table 3) was composed of four Items. Subjects had to rate their level of agreement with each item on an ordinal three-level scale between 1 (disagree) and 3 (agree).

### 2.3. Data Analysis

To evaluate the effectiveness of VIBE in promoting bowing correction, MAE values were used. The presence of any difference between F and NF group during the baseline was assessed with a two-way independent ANOVA. The two factors considered were the group (i.e., F and NF) and the string (i.e., E, A, D, G). The absence of any statistical difference in the baseline between the two groups indicated that they were heterogeneously mixed with similar initial skills. In the experimental phase, a three-way ANOVA was performed on the bowing errors to test the effects of the group, string, and tempi (i.e., 60 bpm, 80 bpm, 100 bpm). Any statistical difference indicated an influence of a factor on the group performance. Finally, to test the effectiveness of the feedback in promoting the behavioral change possibly observed in the experimental phase with respect to the baseline, the bowing performances in the baseline and experimental phases were compared within each group. Since, during the experimental phase, the task was repeated with three different tempi, only the data collected in the most difficult repetition (i.e., 100 bpm) were considered. For this reason, a two-way ANOVA was performed with the protocol phase (i.e., baseline or experimental) and the string as factors. If we observed any difference between phases, this may have been due to the different level of difficulty introduced by the constraint on tempo (in this case, MAE should increase) or the effect of feedback (in this case, MAE should significantly decrease only in the F group).

The choice to use the ANOVA test for performing such comparisons was derived from the fact that it enabled us to evaluate the influence of each single factor on the dependent variable and the interaction between factors separately. This test could be easily applied in this context because each factor had at least two categorical levels. The other test assumptions included the normal distribution of the data, the homogeneity of variance for each factor combination, and the absence of significant outliers. These assumptions were confirmed in the first and third comparisons. In the comparison performed in the experimental phase to test the effect of the group, string, and tempo on the performance, the equality of variances assumption was violated and compensated with a Games–Howell post hoc test.

To assess the usability and the perception of the feedback, SUS and feedback questionnaire scores were exploited. The mean value of the SUS obtained by the F group was compared with data collected on about 1000 SUS [19] to translate the level of usability of the system with an adjective rating scale (i.e., Worst Imaginable, Awful, Poor, Ok, Good, Excellent, and Best Imaginable). The data collected from the questionnaire were used to perform an exploratory analysis of the subjects’ perceptions of the real-time visual feedback.

## 3. Results

**Figure 5:**
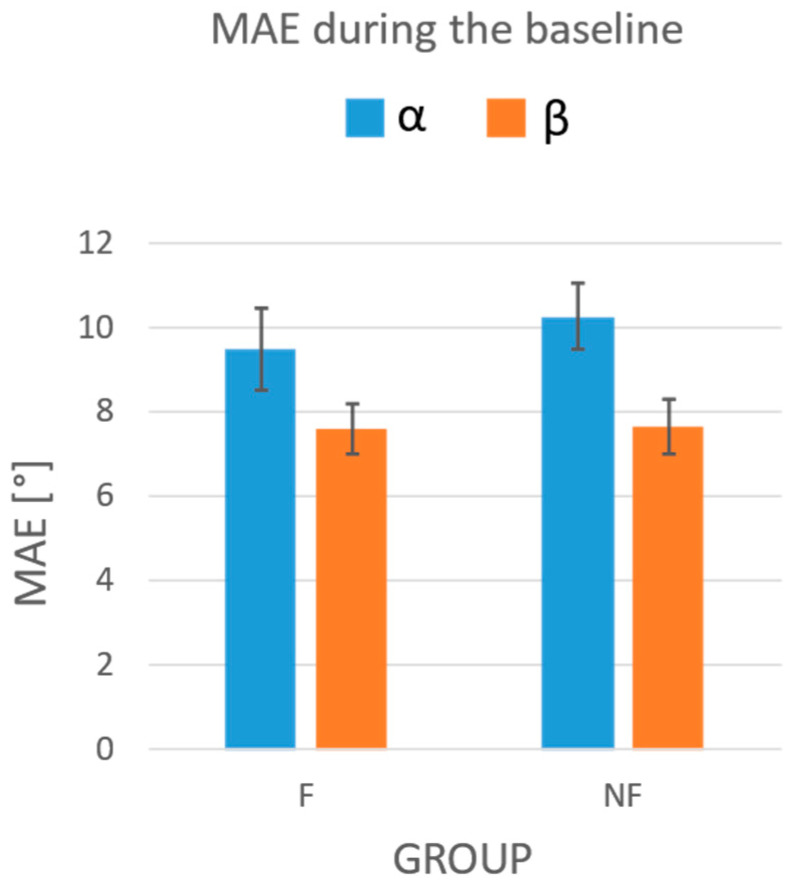
Mean absolute errors obtained during the baseline test in the F and NF groups. In blue: MAEs on the α angle; in orange: MAEs on the β angle. Whiskers represent the standard errors.

The MAEs measured for the F and NF group in the baseline phase are reported in Figure 5. The statistical analysis showed the absence of a statistically significant effect of the condition (F vs. NF) on the errors performed during the baseline, both for the α (*p* = 0.536) and β (*p* = 0.954) angles. The two-way ANOVA also highlighted the absence of a string effect on the MAEs obtained for both bowing angles (*p* = 0.446 for α; *p* = 0.734 for β). The statistical analysis did not show any statistically significant interaction effects between the group and the string for MAE on either the α or β angle (*p* = 0.940 for α; *p* = 0.239 for β).

**Figure 6:**
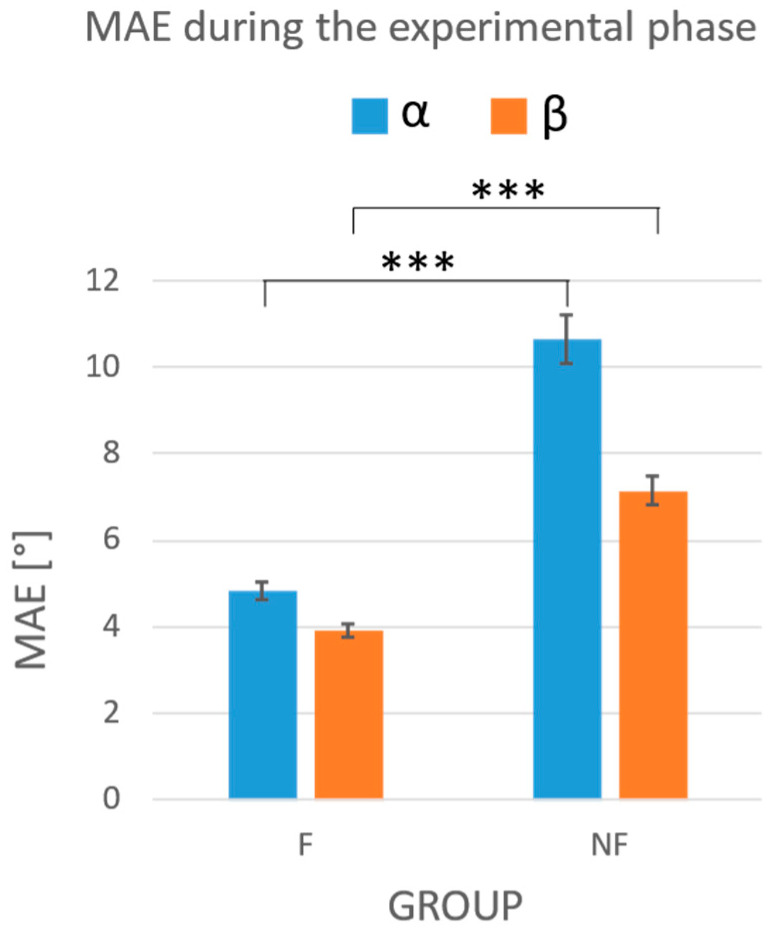
Mean absolute errors obtained during the experimental phase in the F and NF groups. In blue: the MAEs on the α angle; in orange: the MAEs on the β angle. Whiskers represent the standard errors. The statistical analysis showed an effect of the task condition (feedback vs. no feedback) on the reduction in the errors performed by the subjects. The *** indicate a *p*-value less than 0.001.

**Figure 7:**
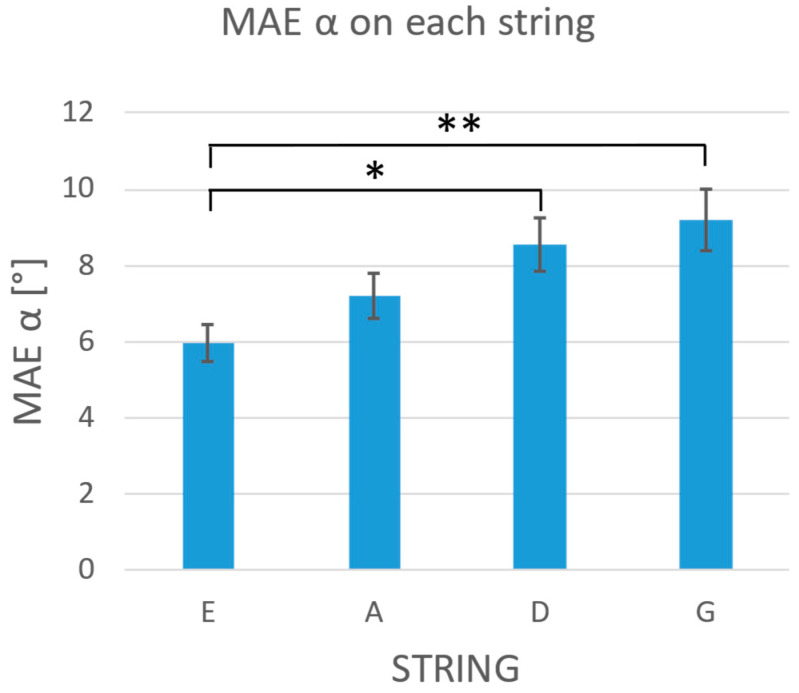
MAE of the α angle on each string. Whiskers represent the standard errors. The statistical analysis highlighted a significant result for the E-D and E-G comparisons. The asterisks in the figure mark statistical significance: * *p*-value < 0.05; ** *p*-value < 0.01.

The results of the three-way ANOVA on the experimental-phase data showed a significant reduction in the MAE in the F group compared to the NF group for both the α (*p* < 0.001) and β angle (*p* < 0.001), as confirmed by the Games–Howell post hoc test (Figure 6). Different tempi did not have any effect on the β angle (*p* = 0.060), but seemed to influence the α angle (*p* = 0.041). The Games–Howell post hoc test did not confirm such an influence for any of the possible tempo comparisons (100 bpm–60 bpm, *p* = 0.190; 100 bpm–80 bpm *p* = 0.080; 60 bpm–80 bpm, *p* = 0.992). Finally, the analysis of the effect of the string on the bowing performance showed a significant effect on the α angle (*p* < 0.001). In particular, the Games–Howell post hoc test showed significantly higher errors of the two distal strings (i.e., D and G) with respect to the proximal one, i.e., the E string (Figure 7, *p* = 0.018 and *p* = 0.004 for D-E and G-E, respectively). The three-way ANOVA did not show any statistically significant interaction between the group, string, and tempi for errors on either α or β angles.

As reported above, the results of the three-way ANOVA highlighted influence of the group on the MAE obtained during the experimental phase both for the α and β angles, suggesting that the introduction of real-time visual feedback promoted a behavioral change in subjects exposed to it.

**Figure 8:**
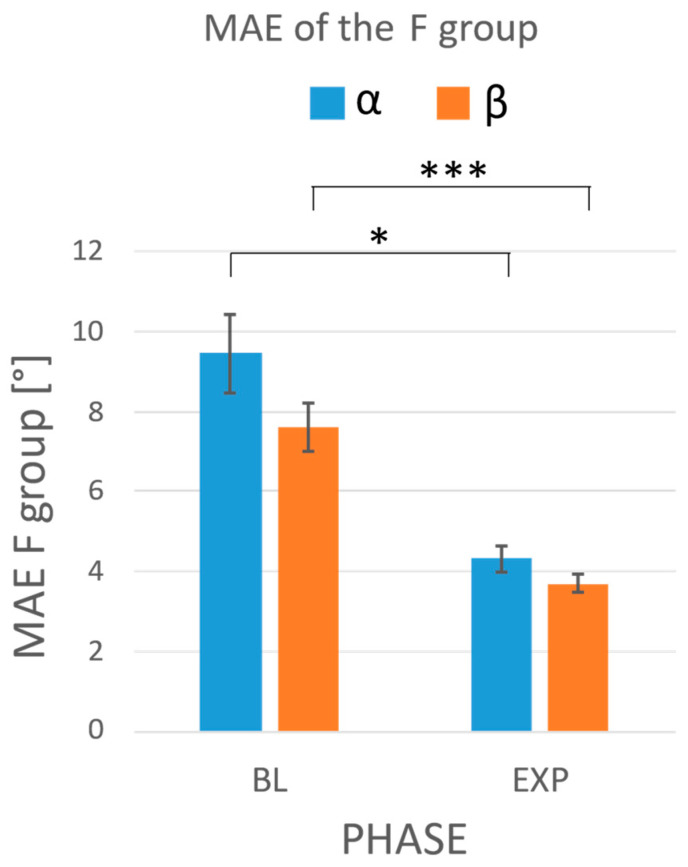
MAEs obtained during the baseline (BL) and the experimental phases (EXP) in the F group. In blue: the MAEs on the α angle; in orange: the MAEs on the β angle. Whiskers represent the standard errors. The statistical analysis showed a significant reduction in the errors performed by the subjects with respect to the baseline. The asterisks in the figure mark statistical significance: * p-value < 0.05; *** *p*-value < 0.001.

**Figure 9:**
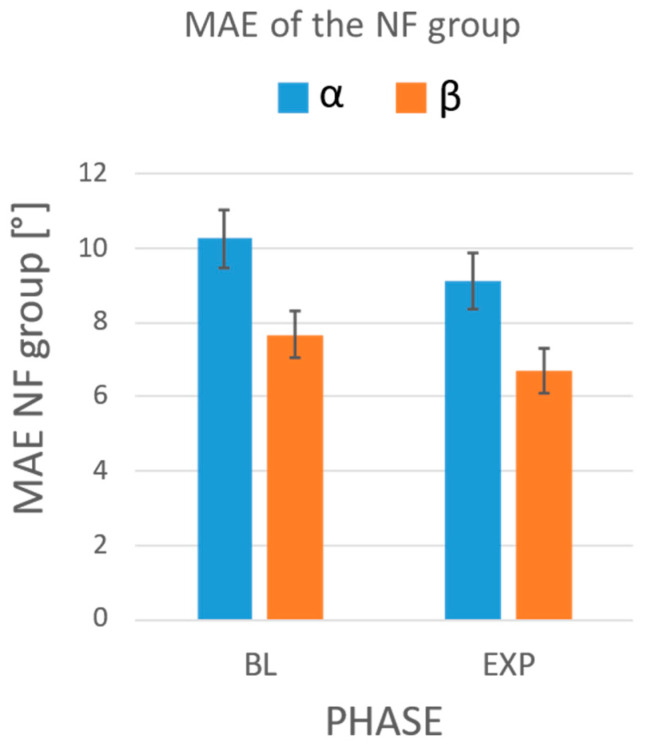
MAEs obtained during the baseline (BL) and the experimental phase (EXP) in the NF group. In blue: the MAEs on the α angle; in orange: the MAEs on the β angle. Whiskers represent the standard error.

The results of the statistical analysis comparing the MAEs of each group between phases showed a significant reduction in the MAEs of the F group in the experimental phase with respect to the baseline, for both the α angle (${MAE}_{BL}=9.47{}^{\circ}\;vs.\;{MAE}_{EXP}=4.32{}^{\circ}$, *p* = 0.012) and the β angle (${MAE}_{BL}=7.61{}^{\circ}\;vs.\;{MAE}_{EXP}=3.70{}^{\circ}$, *p* < 0.001) (Figure 8). The two-way repeated-measure ANOVA did not highlight any influence of the string (*p* = 0.055 for the α angle; *p* = 0.228 for the β angle) or interaction between the two factors (i.e., protocol phase and string, *p* = 0.984 for the α angle; *p* = 0.346 for the β angle) on the MAE. No differences were observed in the NF group (Figure 9).

**Figure 10:**
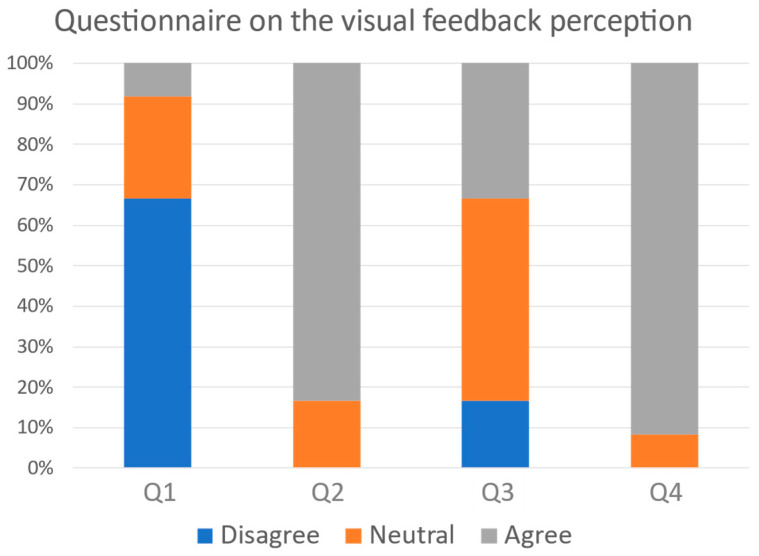
Results of the questionnaire administered to the F group to assess their perception of the visual feedback.

The mean value of the SUS scores obtained from the participants of the F group was equal to 77.29 (SD: 13.46). The comparison with the data collected in [19] revealed that the system’s usability was assessed as excellent. Figure 10 shows the results of the questionnaire on the subjects’ perception of visual feedback: 83.33% of the subjects considered the feedback to be useful to correct their movement, and 91.67% judged the interface to be clear in the presentation of data. Additionally, 66.67% declared that they did not need the presence of a teacher to interpret the feedback, while only 16.67% of the participants found it not to be difficult to perform the task and interpret the feedback at the same time.

## 4. Discussion

The protocol presented in this study aimed at verifying the effectiveness of visual feedback proposed with VIBE and its usability. Previous studies on violin training have already proposed the reconstruction of gesture kinematics to provide learners with different real-time feedback, including vibrotactile [8,10], acoustic [11], and visual [12] feedback. Differently from [8,11], the present study exploits a low-cost technology to reconstruct bowing angles without the necessity of a complex environment. Moreover, these seminal works propose feedback that could influence the musical performance itself (i.e., audio feedback in [11]) or is difficult to interpret in the absence of a teacher (i.e., vibrotactile feedback in [8]). Based on the analysis presented in Table 1, the present work exploits a type of visual feedback judged as one of the most promising among the ones previously adopted. While in [12] only a 2D visual representation of some bowing parameters was provided to users without any information on how to correct the movement, the present study attempts to overcome this limitation by proposing an interface based on MIMUs. This interface not only alerts users to their mistakes, but also suggests how to correct their movements in real-time. While it is true that the mass of MIMU (16 g) impacts the balance of the bow and, consequently, its control by the learner, it is also likely that beginners are not yet sensitive to these differences due to their limited overall control of the bow, as confirmed by our result.

We tested VIBE in a relevant environment with 24 violin beginners: 12 were provided with visual feedback (F group) and 12 were registered without any feedback (NF group). Data gathered in the baseline phase confirmed the heterogeneity of the two groups and their initial skill levels. In the experimental phase, the three-way ANOVA on MAE confirmed the effectiveness of the real-time visual feedback in reducing the errors in the bowing angles (*p* < 0.001 for both angles) and promoting bowing correction, as demonstrated by the comparison of the performance between the baseline and experimental phase. Despite considering the most challenging tempo (i.e., 100 bpm), our data point out a significant MAE reduction in the F group (${MAE}_{BL}=9.47{}^{\circ}\;vs.\;{\;MAE}_{EXP}=4.32{}^{\circ}$, *p* = 0.012 for α and ${MAE}_{BL}=7.61{}^{\circ}\;vs.\;{MAE}_{EXP}=3.70{}^{\circ}$, *p* < 0.001 for β), while data gathered from the NF group do not show any difference. Finally, the three-way ANOVA showed an effect of the string played on the α angle: regardless of the feedback, all participants showed more difficulty in maintaining the bow perpendicular to the string when playing the D and G strings compared to more proximal ones (*p* = 0.018 for E-D comparison; *p* = 0.004 for E-G comparison). This result confirms the preliminary findings reported in [14].

A second key feature of VIBE compared with other works is represented by the technology used to reconstruct the bowing kinematics. While previous works have used optical systems (from complex stereophotogrammetric devices [8,11] to RGB-D cameras [12]), here, we proposed the use of MIMUs to reduce the complexity and cost of the system, thus promoting its usability. For this reason, we asked participants of the F group to fill in the SUS questionnaire, which considered the whole system composed by the MIMUs and the VIBE. The mean reported score was equal to 77.29 (SD: 13.46), which, according to [19], corresponds to an excellent rating.

Finally, the questionnaire on feedback perception enabled the collection of user opinions and the assessment of how the feedback is perceived. The results showed that most of the subjects (91.67%) considered the interface to be clear in the presentation of the feedback, and the majority (66.67%) even considered the presence of a violin teacher not to be necessary for its interpretation. Most of the participants in the experimental group reported that performing the exercise while concurrently interpreting the visual feedback was non-trivial: this result confirms the increase in the cognitive load due to visual feedback, which was already discussed in [12].

These results seem to confirm the good acceptability of the visual feedback, even if the task was perceived as harder. Despite this limitation, the proposed approach respects the requirements described in Section 1, and it is a type of feedback closer to that provided by the teacher during the training (i.e., visual demonstrations).

## 5. Conclusions

Violin teaching, particularly in the initial phases of learning, is primarily based on student–teacher interaction. Teachers play a pivotal role in driving learners to gain awareness of their mistakes. This can be achieved through different possible approaches (verbal instruction, visual demonstration, and physical guidance), which require the continuous presence of the teacher. Unfortunately, this traditional approach cannot be employed during at-home training sessions, which students typically perform alone. This work aims at overcoming this limitation by proposing a novel interface to further support the training of violin beginners even when a teacher is not physically present.

VIBE addresses this goal by exploiting data from two MIMUs on the violin and on the frog to monitor bowing angles and provides students with real-time visual feedback on their gestures. The data collected in this work on 24 beginner violinists confirmed the effectiveness of VIBE in correcting the movements of learners assisted by visual feedback against a control group which did not receive any feedback. Specifically, the experimental group showed a significant reduction in bowing errors. Moreover, despite the additional cognitive load introduced by the visual feedback, the participants considered the overall usability of the system as excellent, the interface clear in its data presentation, the feedback useful to correct their movements, and the presence of a teacher not needed to interpret the feedback.

## Figures and Tables

**Table 1:** The feedback comparison table assesses different feedback reported in the literature according to the criteria defined in the text. For each criterion, a two-level scale is used: Low vs. High for Obtrusiveness, Effectiveness, and Cognitive Load; Requested vs. Not Requested for the necessity of a trained personnel to interpret the feedback.

Feedback	Obtrusiveness	Teacher Presence	Effectiveness	Cognitive Load
Vibrotactile [8,10]	High	Requested	High	High
Audio [11]	Low	Not Requested	Low	Low
Visual [11,12]	High-Low *	Not Requested	High	High

* Depending on the necessity to use markers on the violin beginner. In [11], a stereophotogrammetric marker-based system is used, while in [12], a leap motion is used for markerless reconstruction of the bow.

**Table 2:** β angle target values and their ranges of tolerance.

String	$\beta_{T}\;\pm \;\delta$
G	120 ± 10
D	100 ± 7.5
A	85 ± 7.5
E	65 ± 10

**Table 3:** Questionnaire administered to F group to assess their perception of real-time visual feedback.

Questionnaire about Subjects’ Perception of Real-Time Visual Feedback	
Q1	I thought that I would need the support of a violinist teacher to be able to interpret the visual feedback
Q2	I found the feedback useful to correct my movements
Q3	I found it difficult to execute the exercise and to follow the feedback at the same time
Q4	The interface presented data clearly

## Data Availability

The data presented in this study are available upon request from the corresponding author. The data are not publicly available due to ethical restrictions.
